# Atlanta Academy of Medicine

**Published:** 1871-09

**Authors:** J. T. Johnson


### ATLANTA ACADEMY OF MEDICINE.

REPORTED BY J. T. JOHNSON, M.D.

JULY 14th, 1871.

Dr. Logan in the Chair.

Reports of Sections and Committees being in order, Dr. Love, Chairman of the Section on Gynecology, made a report of a case that had been referred at several meetings previous. The case was one of puerperal convulsions, attended by albuminuria, and occurred in the practice of Drs. Miller and J. M. Johnson. Chloroform had been exhibited *pr. orem*, with the effect of diminishing the amount of albumen in the urine. In reporting the case, Dr. Miller stated that he was in the habit of administering this remedy in the condition named,—that is, albuminuria,—and always succeeded in effecting a diminution, often a total disappearance, of the albumen.

The case had been referred for an investigation of the mode of action of the chloroform—for ascertaining the certainty with which such a result might be expected, and the influence of such result on the course of the disease.

Dr. Love stated that, from various causes, he had been unable to assemble the Section for the consideration of the subject; therefore, would not venture to offer any theories or views of his own in regard to it, but would content himself with giving a full report of the symptoms, treatment and result, as obtained from Dr. Johnson.

(As the case throws no light on the subject as discussed below, and presents nothing new, except as already referred to, it is omitted.)

Dr. H. V. M. Miller was called on for his views in regard to the remedy, and its therapeutical action, as deducted from his experience with it in this case, and similar ones wherein he had used it. Stated that he had but little to say in regard to the case in question; had been so using it for a number of years, as was known, he had supposed, to most or all of the members present. He was first led to this by using it for a lengthened period for the prevention of spasms or convulsions. Observing that it at the same time reduced the albumen, were it present in the urine, he was induced to administer it for the combating of that symptom. When this albuminous condition is ascertained to exist, it should be given for a considerable period three times a day, in doses of twelve or fifteen drops; we thus endeavor to overcome this symptom, and, at the same time, to prevent the convulsions which so often supervene. Whenever we find œdema accompanying pregnancy, we should examine the urine; if albumen is present, resort to chloroform. Writers differ as to the frequency with which puerperal convulsions follow œdema; this difference may originate in the fact of the œdema sometimes arising from other causes than albuminuria—such as pressure on the venous vessels by the uterus, etc. In his experience, chloroform has always effected such a diminution. In the case now considered, it had not been given early enough to prevent the albumen, but had been given only to relieve the convulsions that occurred at the beginning of labor. It proves most efficacious when given from the commencement of œdema.

As to its mode of action, he is not prepared to speak positively. The amount of urine was greatly increased—in some, doubled—in most or all of the cases in which he had used it, differing, in this particular, from the case immediately under discussion. It is possible that it may form a temporary diabetes mellitus, by chemical changes with the albumen. Has experimented with chloral to produce the same result; and though he arrived at nothing definite, it is probable that it may do so, since, as is said, it is decomposed in the blood, and liberates chloroform, one of its original constituents.

Dr. Vance inquires what dose of chloral is requisite for the cure of convulsions; thinks five grains every hour, as ordered in the case discussed, is not sufficient. It seems, too, that the dose was not repeated after the first one. Was witness of a case in which thirty grains were given, and repeated. As to any unpleasant effects consequent upon its use, thinks he saw it tested by the physician in charge of one of the hospitals of France. Passing through the typhoid fever ward, at two o'clock at night, he administered a dose to every one of them, amounting to many in number. There was no nausea or other unpleasant results, and every one took their breakfast the following morning.

Dr. W. F. Westmoreland inquired if Dr. V. regards such doses as were given in the case to which he alludes, as dangerous?

Dr. Vance.—No, if they are given sufficiently diluted; this, the thirty grains, was dissolved in one ounce of glycerine and water.

Dr. J. G. Westmoreland reminds them of the digression from the subject. The question is, if five-grain doses—given instead of chloroform—are large enough to produce the desired change in the urine?

Dr. W. F. Westmoreland.—In this case, the chloral was given, not for albuminuria, but for relief of the convulsions.

Dr. Lowe believes that we should be cautious in the use of chloral. In some diseases, as delirium tremens, there is a wonderful toleration of some medicines otherwise active. Such may have been the case in some of the instances referred to by Dr. Vance.

Dr. Vance had seen a case, in a hospital, in which seventy-five grains were given within an hour. When the last portion of twenty-five grains was given, the patient fell as suddenly as if shot, but awoke all right in an hour.

Dr. W. A. Love, questioned by Dr. W. F. Westmoreland, repeats that he only gave a history of the case, not venturing to offer any opinion or theory of his own as to the action of chloroform, since he was not able to assemble the Section, and was aware that he differed from some of the members composing it. He is compelled to differ with Dr. Miller as to the mode in which chloroform affects this change in the urine. In the first place, thinks the albumen in the blood results from a deficiency of oxygenation—this deficiency of respiration being caused by a spasm of the bronchial tubes. Chloroform, or some similar remedy, relaxes this spasm, and thus makes to disappear the excess of albumen in the blood.

Dr. Miller asks if Dr. Love would assert that the *blood* of a pregnant woman, or other person, affected with albuminuria, contains more albumen than in health?

Dr. Love believes that it *is* increased.

Dr. Miller is aware that there is a definite proportion of albumen in the blood of a healthy person, but believes that the increase of this substance in the urine of albuminuria is attended with its waste, rather than otherwise, in the blood. Though he has examined the record of many analyses, made by many observers, of the blood in this disease, has not yet met with one showing the condition that Dr. Love holds to be characteristic. While he may be exposing his own ignorance, is willing so to do if thereby he can obtain information of a condition which he has thought never exists.

Dr. Love replies that he is confident of having seen such an analysis,—cannot be positive as to where, but yet is sure of having seen it; will find it, and show it to Dr. Miller. Would add, that he long since used chloroform in cases of intermittent albuminuria, existing for an hour or two during the twenty-four, which he had met in some diseases attended by congestion.

Dr. W. A. Cumming moves, that as this is an important matter, not only as to the condition of the blood in albuminuria, but as to the certainty claimed for the influence of chloroform in producing the change in the urine, and the manner of the working of that change, it be again committed to the Section on Gynecology, for further consideration and report.

Seconded and carried.

REPORTS OF CASES.

Dr. J. T. Johnson reports a case—since chloral has excited some interest in the meeting—in which he had used that remedy, in delirium tremens, in conjunction with morphia. The case was a decided one, though it had not reached its most confirmed stage. Gave thirty-five grains of the chloral, and a hypodermic injection of one-fifth of a grain of sulphate of morphia at the same time. This produced a slight sleep, but the patient was disturbed. In four hours, thirty more grains of the chloral were administered, followed by many hours' sleep, with relief from all symptoms. To prevent that local irritation which is feared by some, dissolved it in six ounces of water.

QUESTION FOR DISCUSSION.

The appointed subject was in reference to the "Antidotal Action of Opium and Belladonna," but the lateness of the hour rendered adjournment necessary.

(*Extract from Minutes, July* 21, 1871.)

Dr. J. G. Westmoreland reports some cases of fever, of an unusual type, that have recently occurred in his practice. It bears some resemblance to both typhoid and remittent, and yet is unlike either. The fever is not continued, but presents a distinctly remitting character—the remissions occurring with regularity, and usually in the morning. Still, the remission is not as well-marked as in remittent fever. Antiperiodics, as quinine, have no control over its course or duration, though it was thoroughly tested. There was redness of the tongue in all of the three cases, and in one, diarrhea. The duration of the fever is two weeks—differing, in that respect, from typhoid, which lasts three weeks, and from remittent, which terminates, usually, in one week. But it differs from the latter, especially, in that it is not at all amenable to quinine. In the year 1847, in the country in which he was then practicing, there was an epidemic of the same character, extending over the whole country; then some few of the cases died, but the rule was recovery. Again, in 1866, there appeared in Atlanta an epidemic of the same fever. The cases now reported seem to be altogether similar to those epidemics. Two of these cases have recovered; the third is convalescing.

Dr. Wells.—Is there headache present?

Dr. Westmoreland.—Yes; also a stiffness and soreness of the back of the neck.

Dr. Wells.—Any tympanites?

Dr. Westmoreland.—None, though in one there was a persistent diarrhea.

Dr. Judson.—What are the first symptoms?

Dr. Westmoreland.—Uneasiness and lassitude for several days; also, the soreness of the neck already alluded to.

Dr. Wilson.—What were the premonitory symptoms?

Dr. Westmoreland.—Several days were consumed by the symptoms just mentioned. The patients all have been able, even when at the worst, to sit up occasionally in bed or in a chair. Used, in addition to the twenty grains of quinine, a purgative or two, and a blister to the back of the neck.

Dr. Wells.—Did you give any sedative?

Dr. Westmoreland.—No; there was no indication for it, though the pulse was somewhat frequent.

Dr. Wells thinks we are too much in the habit of regarding typhoid fever as of necessity a severe, if not a fatal disease. During some epidemics, it is more amenable to treatment than in others. Cases may recover before the expiration of the allotted three weeks,—that is, unless we include the premonitory stages as a part of the disease. Dr. Westmoreland's cases, he doubts not, are instances of genuine typhoid fever. In typhoid, we may have some remission during the morning, especially if malaria is prevalent in the neighborhood.

Dr. J. G. Westmoreland has seen a great deal of typhoid fever—especially in the years 1850 to 1860—and never yet saw one recover in less than three weeks.

Dr. Wells is sure that it sometimes does not, by one week, last so long as twenty-one days. Would add, that, as to its origin, he does not believe in the blood-poisoning theory; regards the nerve-centers as the prime seat of trouble.

Dr. W. H. Cumming.—There has been nothing connected with hygiene so interesting as the observations in England, for the last few years, in reference to the cause of typhoid fever. It has been almost fixed, in many cases, on water contaminated with fæces. In one street, in which the opposite sides were supplied with water from different sources, the inhabitants of one side suffered from its depredations, while the other escaped entirely. The conviction is growing stronger, that this is one of the causes of typhoid fever. Would ask Dr. Westmoreland if the persistent debility characteristic of typhoid fever was present in the cases he has reported, and if it was present in the same disease as observed by himself in former years?

Dr. Westmoreland.—The debility is not present.

Dr. W. N. Judson has observed cases that agree in character with those reported. One of the patients was unable to leave the bed for two weeks; in the other, there was high delirium on the third day, with great irritability of the nervous system. Treated one of the cases for five weeks, though there was sometimes an entire absence of fever; but it would usually come on about six o'clock P. M. Could find no treatment that seemed to benefit it.

Dr. Wilson has met none of this fever in his practice. Does not believe that typhoid fever always lasts three weeks,—has known it to terminate in two weeks. Has seen cases of mongrel fever that resembled both typhoid and remittent. Would discard the theories that locate typhoid fever primarily in the blood or nervous system; adheres to the French pathology, that the affected intestinal glands are the first part to suffer.

Dr. W. F. Westmoreland has seen only one case, recently, of this unusual type of fever. It is evidently the same as that which prevailed in this city in 1866 and 1867, and that was the first he had seen in twenty years. It differs from both typhoid and remittent, especially as regards the tongue and the exacerbation. Sometimes the patient is exempt from fever for four out of the twenty-four hours. While it is probably not of malarial origin, yet he has never observed it unless there was at the same time a prevalence of malaria. In this city, its appearance has coincided with that of intermittent fever. The city has been, until recently, for a long while free from intermittent fever, but learns that it is again prevailing to some extent. As to the nature of typhoid fever, he believes that it is essentially an eruptive disease,—one as truly exanthematous as is small-pox,—a disease of the skin and mucous membranes. But he has never been able to satisfy himself as to the question of its contagiousness. We do not always appreciate the influence of epidemics in impressing their character on all diseases that may chance to exist within the range of their influence. Merely the character of this disease, not its seat, is expressed by the name "typhoid." Other diseases may be typhoid. We may have typhoid dysentery, pneumonia, remittent fever, or a typhoid broken leg. Remembers well the disposition of the epidemic of 1851 and 1852 to lend its coloring to all diseases whatsoever.

Dr. J. P. Logan has seen, in the last two weeks, several cases of this peculiar fever. Though it distinctly remits, it is not at all controlled by quinine; indeed, if this remedy exerts any influence at all, it is rather an aggravation of the symptoms. He was led to abandon its use entirely, and resort to veratrum, anodynes, etc., but doubts if any results were obtained by the use of medicine. As observed by himself, it is identical with the disease described by Dr. Westmoreland. It terminates in fourteen days, and is not malarious, as he is fully satisfied from close observation. Thinks he has seen a double paroxysm in the twenty-four hours.

Dr. J. G. Westmoreland says this is the third time he has come in contact with this fever. For the sake of distinction, as it has always borne a well-marked type, has called it "the two weeks' fever."

Dr. Cumming reminds him that this name has been already assigned to typhus fever, to distinguish it from the "one week fever" (bilious remittent) and the "three weeks'" (typhoid.)

Dr. H. V. M. Miller, calling attention to the wide range the subject has taken, asks the opinion of the members as to the difference between this fever and that known during the war as "camp fever." As described by Dr. Westmoreland, thinks it is similar to this. Would inquire, further, as to what is the nervous center affected in typhoid? And what is the evidence of such affection? Remembers that quinine was of no value in camp fever. But few died from the disease. This is not to be confounded with the malarial fever of the peninsular campaign of Virginia.

Dr. J. G. Westmoreland had much experience with the fevers of the army. Saw intermittent and remittent, as well as camp fever. If he ever saw a case of typhoid fever, the last is undoubtedly that disease. There was no variation from it; there was the same nervous depression, the same course, the intercurrent bronchitis, and the same duration. Saw no recovery short of three weeks. As to Dr. Miller's question, as to what is the "nervous center" affected, would reply, that the encephalon is first involved, as indicated by the symptoms. Has frequently diagnosed a case by the presence or absence of the symptoms pertaining to that organ. The mucous membrane of the bowels or lungs may be affected, resulting secondarily from this original affection of the brain.

Dr. W. F. Westmoreland, having had much experience in camp fever at Centreville, Virginia, in Mississippi, and in this place in 1864, must pronounce it very different from typhoid; its duration was only ten or fifteen days. As to which is the primary seat of trouble in typhoid fever, we can not, merely because of the nervous symptoms, infer that the brain is first diseased. We may have the same phenomena in small-pox, for instance, and in many other diseases. There is not always a lesion of the brain, as shown by post-mortem; but the lesion of the mucous membrane of the bowel is always present, even when not indicated by diarrhea before death.

Dr. J. G. Westmoreland.—We may have the mucous membrane of the bronchia diseased in some cases; in others, not. In the latter case, we will probably have involved the mucous membrane of some portion of the alimentary canal, as discovered by the red tongue. Again, in other cases, we have neither of the membranes involved. These variations show that disease of none of these tissues is an essential part of the disease, but that all result from the primary disease of the nervous system. We find further proof in the fact that we succeed best with those remedies that address themselves to that system. The nervous energy is thus sustained by such remedies as alcohol, opium, etc.

Dr. W. F. Westmoreland.—Dr. J. G. Westmoreland assumes that there are cases in which exists no ulceration of the glands of the mucous membrane of the intestine. Must insist that absence of diarrhea is no guarantee of non-ulceration. Even perforation may occur without there having been any disturbance of the bowels. With an extensive experience in autopsical examination, he never saw a case in which the lesions of the bronchial mucous membranes were wanting. This disease of the mucous membrane is of the same nature as the eruption on the surface of the body.

Dr. J. S. Wilson.—If there is a lesion of the nervous centers, it must occur through the blood. The lesion of the glands is also superinduced secondarily. If the eruption, as alluded to by Dr. W. F. Westmoreland, is an essential characteristic of typhoid fever, then he has seen but little of the disease. In fact, he has never witnessed it in as many as six cases, and must regard it as only an accidental complication. The blood produces the nervous symptoms; it yet remains for us to assure ourselves of the cause of the blood disease.

Dr. H. V. M. Miller believes in none of the theories that have been advanced; but as it would require much time to give his many disbeliefs, and his reasons therefor, will not so encroach on the time of the Academy.

Adjourned.

